# lncRNA CCAT1 promotes bladder cancer cell proliferation, migration and invasion

**DOI:** 10.1590/S1677-5538.IBJU.2018.0450

**Published:** 2019-07-27

**Authors:** Caixiang Zhang, Wenying Wang, Jun Lin, Jing Xiao, Ye Tian

**Affiliations:** 1 Department of Urology, Beijing Friendship Hospital, Capital Medical University, Beijing, China

**Keywords:** Urinary Bladder Neoplasms, CCAT1 long noncoding RNA, human [Supplementary Concept], Cell Proliferation

## Abstract

**Objective:**

To study the expression patterns of long noncoding RNA (lncRNA) colon cancer-associated transcript 1 (CCAT1) and the changes in cell proliferation, apoptosis, migration and invasion induced by silencing CCAT1 in bladder cancer cells.

**Materials and Methods:**

The expression levels of CCAT1 were determined using realtime quantitative polymerase chain reaction in cancerous tissues and paired normal tissues from 34 patients with bladder cancer. The relationship between clinical characteristics and CCAT1 expression was analyzed. And then we conducted cell experiments. Bladder urothelial carcinoma cell lines T24 and 5637 cells were transfected with CCAT1 small interfering RNA (siRNA) or scramble siRNA. Cell proliferation and apoptosis changes were determined using a Cell Counting Kit-8 (CCK-8) assay and a flow cytometry assay. Migration and invasion changes were measured using a wound healing assay and a trans-well assay. microRNAs (miRNAs) were predicted by Starbase 2.0, and their differential expression levels were studied.

**Results:**

CCAT1 was significantly upregulated in bladder cancer (P < 0.05). CCAT1 upregulation was positively related to tumor stage (P = 0.004), tumor grade (P = 0.001) and tumor size (P = 0.042). Cell proliferation, migration and invasion were promoted by abnormally expressed CCAT1. miRNAs miR-181b-5p, miR-152-3p, miR-24-3p, miR-148a-3p and miR-490-3p were potentially related to the aforementioned functions of CCAT1.

**Conclusion:**

CCAT1 plays an oncogenic role in urothelial carcinoma of the bladder. In addition, CCAT1 may be a potential therapeutic target in this cancer.

## INTRODUCTION

Bladder cancer is the ninth most common cancer worldwide (1). Most malignant bladder tumors are urothelial carcinomas evolved from the urothelium. Approximately 40% of patients with bladder cancer have experienced multiple recurrences, significantly impacting their life quality (2). Bladder cancer is a heterogeneous disease that can be categorized into non-muscle invasive bladder cancer and muscle invasive bladder cancer. Urothelial carcinomas could be divided into low-grade and high-grade urothelial carcinomas. Muscle invasive or high-grade bladder tumors are more aggressive relatively (3). And tumors greater than 3cm comprise a risk factor of recurrence and progression (4). The mortality of bladder cancer has not changed markedly despite recent advances in its early detection (5). Therefore, it is necessary to delineate the molecular mechanisms involved in the development of bladder cancer to find potentially novel and important diagnostic markers and therapeutic targets.

Noncoding RNAs (ncRNAs) are subdivided into two major classes based on size: small ncRNAs (< 200nt) and long ncRNAs (lncRNAs, > 200nt) (6, 7). Recently, microRNAs (miRNAs) have been identified as oncogenes or tumor suppressor genes that influence the biological function of cancer through the posttranscriptional regulation of protein expression. In contrast, lncRNAs were once thought to be transcriptional noise (8). However, it has become increasingly clear that lncRNAs exert significant functions at various levels, including X chromosomal inactivation, chromatin remodeling and transcriptional repression. In addition, studies have found that some lncRNAs are contained in the urinary exosomes of bladder cancer patients, which is important for alleviating the suffering of patients who require regular postoperative cystoscopy (9).

The lncRNA CCAT1, which is abnormally expressed in many malignancies, has been reported to be involved in the pathogenesis of malignant tumors, including colon cancer, oral squamous cell carcinoma, epithelial ovarian cancer, renal cell carcinoma, hepatocellular carcinoma, cervical cancer, breast cancer, gallbladder cancer and gastric cancer (10–13). Further studies have found that CCAT1 transcription was stimulated by c-Myc, which then promoted the proliferation and invasion of cancer cells (14, 15). Other studies have found that CCAT1 promotes cancer development via miRNAs (16, 17). CCAT1 has been used as a novel serum biomarker in colorectal carcinoma (18). However, the role of CCAT1 in the development of bladder cancer remains elusive.

## MATERIALS AND METHODS

### Patient samples

In the current study, 34 consecutive individuals with urothelial neoplasms of the bladder who had received radical cystectomy were included. Bladder cancer tissues and pair-matched adjacent healthy tissues were snap-frozen in liquid nitrogen within thirty min after resection, and the upregulation rate of CCAT1 was analyzed. All patients had negative histories of exposure to either chemotherapy or radiotherapy before surgery and lacked the co-occurrence of any diagnosed cancers. This study was approved by the Ethical Committee of Beijing Friendship Hospital Affiliated with Capital Medical University.

### Cell lines and cell culture

Human bladder cancer cell lines (T24, EJ, and 5637) were purchased from National Infrastructure of Cell Line Resource (Beijing, China). The T24, EJ and 5637 cells were cultured in RPMI-1640 medium (Gibco, USA) plus 1% antibiotics (100U/mL penicillin and 100μg/mL streptomycin sulfate) and 10% fetal bovine serum (FBS, Gibco) at 37°C in a humidified atmosphere with 5% CO_2_. Complete medium was changed every 2 days, and cells were subcultured when they reached 80% confluence.

### RNA extraction, reverse transcription and qRT-PCR

Total RNA was isolated from bladder cancer tissues or cells using TRIzol reagent (Invitrogen, Carlsbad, CA, USA) according to the manufacturer's protocol to measure relative CCAT1 expression. Reverse transcription was then performed using a Prime ScriptTM RT Reagent Kit with gDNA Eraser (Takara, Dalian, China) following the manufacturer's instructions. Quantitative real-time polymerase chain reaction (qRT-PCR) was performed using a SYBR Green PCR kit (Takara, Dalian, China) following the manufacturer's instructions. Glyceraldehyde 3-phosphate dehydrogenase (GAPDH) was measured as an internal control. The reactions were carried out on an ABI^®^ 7500 Fast Real-Time PCR system (Applied Biosystems, Foster City, CA, USA) in duplicate. The reaction conditions were 95°C for 10 min, 40 cycles at 95°C for 15 sec and 60°C for 35sec. The melting curve was analyzed for each sample. The average value in each duplicate was used to calculate the relative amount of CCAT1 using the 2^−ΔΔCt^ method. The primer sequences were as follows: CCAT1 primers: 5’-TTTATGCTTGAGCCTTGA-3’ (forward) and 5’- CTTGCCTGAAATACTTGC-3’ (reverse) and GAPDH primers: 5’-CGCTCTCTGCTCCTCCTGTTC-3’ (forward) and 5’-ATCCGTTGACTCCGACCTTCAC-3’ (reverse).

miRNA expression was detected by extracting miRNA from cell lines using a miRcute miRNA isolation kit (Tiangen, Beijing, China) according to the manufacturer's protocol. Quantified total RNA was then reverse transcribed to cDNA using a miRcute Plus miRNA First-Strand cDNA Synthesis Kit (Tiangen, Beijing, China) following the manufacturer's instructions. qRT-PCR was conducted using miRcute Plus miRNA qPCR Detection Kit (SYBR Green) (Tiangen, Beijing, China) following the manufacturer's instructions. Transcripts were quantified by qRT-PCR and normalized to the amount of U6 mRNA expression. The reactions were carried out on an ABI^®^ 7500 Fast Real-Time PCR system (Applied Biosystems, Foster City, CA, USA) in duplicate under the following conditions: 95°C for 15 min, 40 cycles at 95°C for 20sec, and 60°C for 35sec. The melting curve was analyzed for each sample. The average value in each duplicate was used to calculate the relative amount of miRNA using 2-^ΔΔCt^ methods. The primer sequences of U6 were as follows: 5’-CTCGCTTCGGCAGCACA-3’ (forward) and 5’-AACGCTTCACGAATTTGCGT-3’ (reverse), and the primer sequences of all miRNAs are listed at supplemental Table-1.

**Supplemental Table 1:** The primer sequences of all the miRNAs.

microRNA name	primer sequences
hsa-miR-181b-5p	ccgAACATTCATTGCTGTCGGTGGGT
hsa-miR-181a-5p	cgcgAACATTCAACGCTGTCGGTGAGT
hsa-miR-4295	cgcgcgCAGTGCAATGTTTTCCTT
hsa-miR-130a-3p	gcgcgCAGTGCAATGTTAAAAGGGCAT
hsa-miR-148b-3p	gcgcgTCAGTGCATCACAGAACTTTGT
hsa-miR-543	cgcgAAACATTCGCGGTGCACTTCTT
hsa-miR-410-3p	gcgcgcgAATATAACACAGATGGCCTGT
hsa-miR-152-3p	gcgcgTCAGTGCATGACAGAACTTGG
hsa-miR-454-3p	agcgcgcgTAGTGCAAWTGCTTATAGGGT
hsa-miR-301a-3p	cgcgcgCAGTGCAATAGTATTGTCAAAGC
hsa-miR-24-3p	gcgTGGCTCAGTTCAGCAGGAACAG
hsa-miR-181c-5p	gcgcAACATTCAACCTGTCGGTGAGT
hsa-miR-181d-5p	cgcgAACATTCATTGTTGTCGGTGGGT
hsa-miR-4262	gcgcgcgGACATTCAGACTACCTG
hsa-miR-216a-5p	ccgcgTAATCTCAGCTGGCAACTGTGA
hsa-miR-296-3p	GAGGGTTGGGTGGAGGCTCTCC
hsa-miR-301b	gcgGCTCTGACGAGGTTGCACTACT
hsa-miR-130b-3p	cgcgCAGTGCAATGATGAAAGGGCAT
hsa-miR-218-5p	ccgcgcgTTGTGCTTGATCTAACCATGT
hsa-miR-148a-3p	cgcgcgTCAGTGCACTACAGAACTTTGT
hsa-miR-3666	gcgCAGTGCAAGTGTAGATGCCGA
hsa-miR-490-3p	cgCAACCTGGAGGACTCCATGCTG

### Small interfering RNA (siRNA) synthesis and transfection

siRNA specifically targeting CCAT1 and scramble siRNA were synthesized by GenePharma (Shanghai, China). The siRNA sequences for CCAT1 were si-CCAT1-1, 5′-CAUACCAAUUGAACCGAGCCUUGUA-3′, and si-CCAT1-2, 5′-CCATTCCATTCATTTCTCTTTCCTA-3′. Transfections were performed using the Lipofectamine 2000 kit (Invitrogen, Carlsbad, CA, USA) according to the manufacturer's instructions.

### Cell proliferation assays

Cell proliferation was determined using a Cell Counting Kit-8 (CCK-8, Beyotime Institute of Biotechnology, Shanghai, China) according to the manufacturer's instructions. For the CCK-8 assay, 2 × 10^4^ cells/well were seeded in a 96-well plate for 24h and were transiently transfected with si-CCAT1-1, si-CCAT1-2 or scramble siRNA. Ten microliters of the Cell Counting Kit solution was added into each well at 0, 1, 2 and 3 days after transfection. The 96-well plates were incubated for 2h at 37°C, and the absorbencies at each time point were measured at 450nm by a microplate reader. All experiments were biologically repeated at least three times.

### Wound healing assays

Cells (1.25 × 10^5^ cells/well) were seeded into 24-well plates and starved for 24h; then, the medium was replaced with medium containing 10% FBS. Wounds were made by passing a plastic tip across the monolayered cells. The time of wound infliction was considered as 0h, and wound closure was photographed after 48h using a microscope connected to a digital camera. Areas covered by migrated cells (%) were quantified by ImageJ 1.48v. All experiments were performed in triplicate.

### Trans-well assay

Cells transfected with si-CCAT1-1, si-CCAT1-2 or scramble siRNA were first starved in serum-free medium, and then, a cell suspension of 0.2mL of RPMI-1640 medium was seeded into each well of the upper trans-well chamber (8-pm pore size, Corning, New York, USA), which was precoated with 50μl of Matrigel™ Basement Membrane Matrix (BD Biosciences, San Jose, CA, USA). In the lower chamber, 0.5mL of RPMI 1640 with 10% FBS was added. After incubating for 28h at 37°C in a humidified incubator with 5% CO_2_, the cells on the upper side of the upper chamber were wiped off with a cotton swab. The lower side of the upper chamber was fixed with methanol and stained with 2% crystal violet for 10 min. The number of cells penetrating across the membrane was counted under a microscope in five random visual fields, and all experiments were repeated in triplicate.

### Flow cytometry assay

T24 and 5637 cells were transfected with si-CCAT1-1, si-CCAT1-2 or scramble siRNA. After 48h, the cells were harvested for flow cytometry. Briefly, after double staining with an Annexin V-FITC/PI apoptosis kit (Multi Sciences, Hangzhou, China) according to the manufacturer's instructions, cell apoptosis was then determined using a flow cytometer (BD, NJ, USA). All experiments were performed in triplicate.

### Statistical analyses

All data were analyzed by SPSS 22.0 software. Values were expressed as the mean ± standard deviation (SD). A paired t-test was used to analyze the CCAT1 expression difference between bladder cancer tissues and paracancer tissues. A chi-square test or unpaired Student's t-test was also used to evaluate significance as appropriate. P < 0.05 was considered significant.

## RESULTS

### CCAT1 is upregulated in bladder cancer tissues

**Figure 1:**
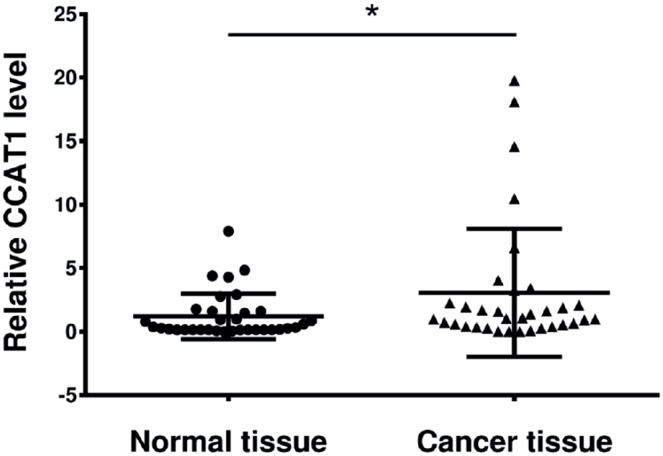
The gene expression level of CCAT1 in human bladder tumor tissues and paratumor tissues (n = 34). Data are presented as mean ± SD. *Indicates P < 0.05 versus the control group.

First, we investigated whether CCAT1 was dysregulated in bladder cancer tissues relative to its expression in adjacent healthy cells. The relative expression level of CCAT1 was measured using qRT-PCR in bladder cancer tissues and pair-matched adjacent normal bladder tissues from 34 bladder cancer patients. Compared to that in pair-matched adjacent normal bladder tissues, CCAT1 expression was upregulated significantly in 73.53% (25 of 34) of cancer tissues (Figure-1, P<0.05).

### CCAT1 expression is associated with clinicopathological features

We investigated the relationship between increased CCAT1 expression levels and clinical characteristics in the 34 bladder cancer cases to determine whether CCAT1 expression is related to clinical features (Table-1). CCAT1 upregulation was positively correlated with tumor stage (P=0.004), tumor grade (P=0.001) and tumor size (P=0.042). However, CCAT1 expression levels were not correlated with other parameters, such as patient age, gender, tumor number or nodal invasion. Bladder cancer tumors were graded according to the 2004WHO classification of urothelial neoplasms.

**Table 1:** Relationship between CCAT1 expression and clinicopathological characteristics of bladder cancer patients.

Characteristics		No.	CCAT1 expression level		P-value
			Downregulation	Upregulation	
**Age (year)**					
	≤65	13	4	9	0.679
	>65	21	4	17	
**Gender**					
	Male	22	7	15	0.21
	Female	12	1	11	
**Tumor staging**					
	<2	12	6	6	0.004
	≥2	22	1	21	
**Tumor grading**					
	Low grade	7	5	2	0.001
	High grade	27	2	25	
**Tumor size (cm)**					
	≤3	14	6	8	0.042
	3	20	2	18	
**Tumor amount**					
	Single	13	2	11	0.444
	Multiple	21	6	15	
**Nodal invasion**					
	Positive	2	1	1	0.421
	Negative	32	7	25	

### The effects of CCAT1 knockdown on bladder cancer cell lines

**Supplemental Figure 1:**
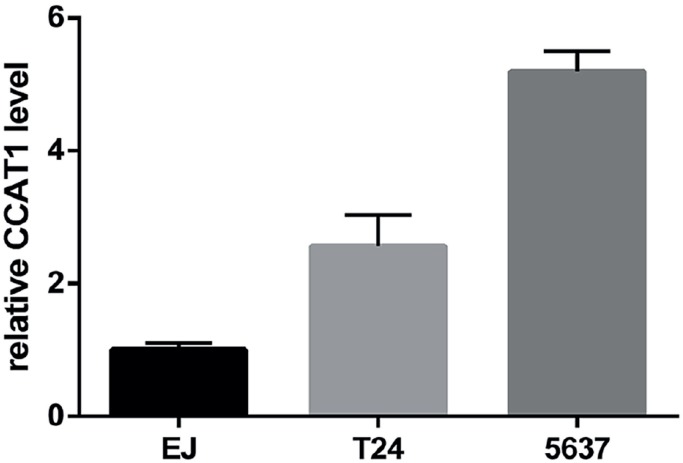
The expression level of CCAT1 in EJ, T24, 5637 cell lines.

**Figure 2:**
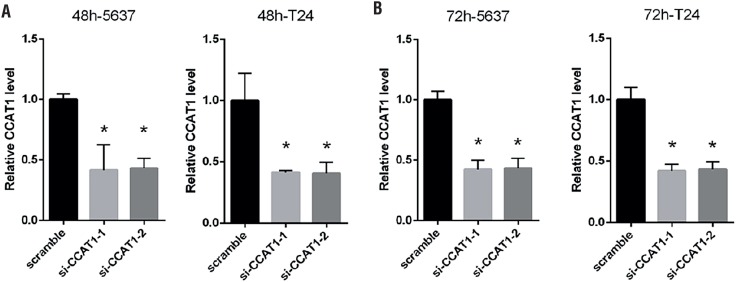
The gene expression level of CCAT1 knockdown on 5637 and T24 cell lines 48 hours or 72 hours after transfection. Data are presented as mean ± SD. *Indicates P < 0.05 versus the control group.

As differences in tumor stage, grade and size may be derived from tumor cell functions, we first developed an in vitro model of CCAT1 knock-down bladder tumor cells. CCAT1 expression in three human bladder cancer cell lines (EJ, T24, and 5637) was initially investigated (supplemental Figure-1). T24 and 5637 cells showed relatively high CCAT1 levels and were used in our CCAT1 siRNA knock-down system.

si-CCAT1-1, si-CCAT1-2 or scramble siRNA was transiently transfected into 5637 and T24 cells to assess the effects of CCAT1 on bladder cancer cells. 48 hours and 72 hours after transfection, qRT-PCR was performed and the result demonstrated that the relative CCAT1 expression in CCAT1-siRNA-transfected 5637 and T24 cells was significantly lower than the relative expression in the negative control group (P<0.05; Figures 2A and 2B).

**Figure 3:**
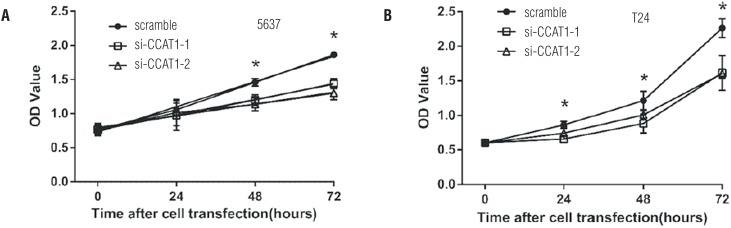
Effects of CCAT1 on proliferation in 5637 and T24 cell lines. CCK-8 cell proliferation assays show that CCAT1 knockdown significantly weakened proliferation in 5637 and T24 cells. Data are represented as mean ± SD. indicates P < 0.05.

**Supplemental Figure 2:**
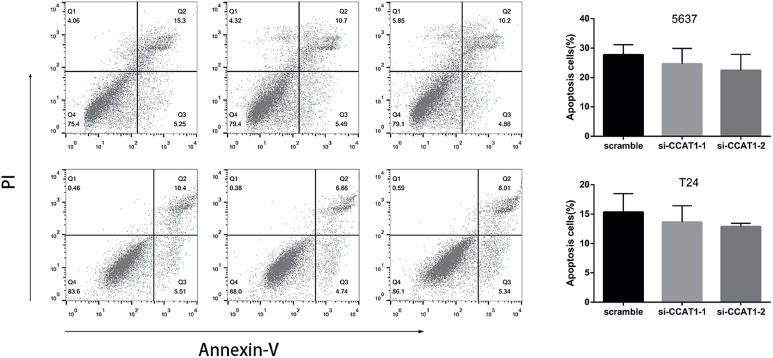
Representative flow cytometric plots of cell apoptosis. Cells were stained with both Annexin V and PI before analysis by flow cytometry. Numbers represent the percentage of the frequency in each quadrant. Data are presented as mean ± SD. *Indicates P < 0.05

**Figure 4:**
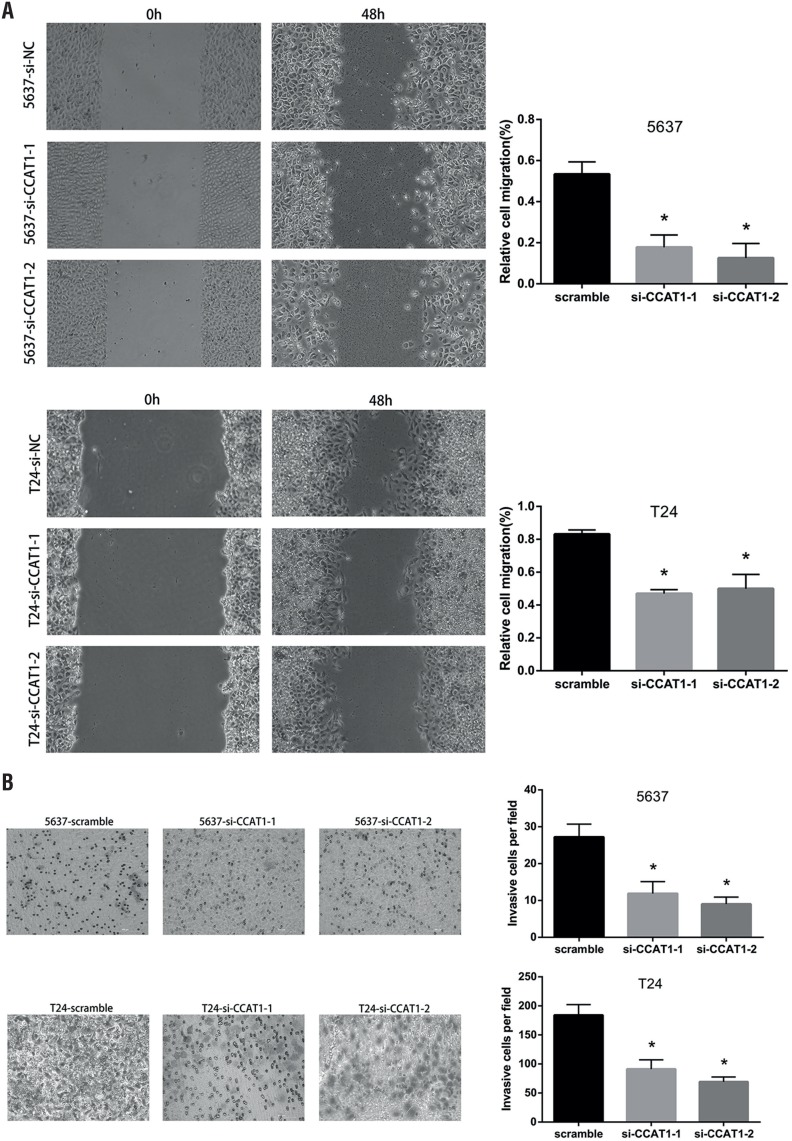
Effects of CCAT1 on migration and invasion capability in 5637 and T24 cells. A) Knock-down of CCAT1 attenuated the migration ability of 5637 and T24 cells. The quantifications of cell migration were presented by the histogram. B) Transwell assay indicated that knock-down of CCAT1 weakened 5637 and T24 cells invasion. The quantifications of cell invasion were presented by the column chart. Data are presented as mean ± SD. *Indicates significant difference compared with control group (P<0.05).

To determine whether CCAT1 was associated with cell functions, we then compared the proliferation, apoptosis, migration and invasion of CCAT1-siRNA knock-down cells and scramble siRNA-infected bladder cancer cells. The CCK-8 assays indicated that CCAT1-siRNA treatment significantly inhibited the proliferation of 5637 cells at 48h and 72h post-transfection and T24 cells at 24h, 48h and 72h post-transfection (Figure-3). Flow cytological examination was performed after the double staining of Annexin V-PI to examine the effect of CCAT1 on apoptosis. We found that the downregulation of CCAT1 had no effect on apoptosis in bladder cancer cells by analyzing the percentage of cells in the third quadrant (supplemental Figure-2). We performed wound healing assays to assess the bladder cancer cell's ability to migrate in the horizontal direction. Knock-down of CCAT1 attenuated the migration abilities of 5637 and T24 cells according to an analysis of the area covered by cell migration at 48h post-transfection (P<0.05, Figure-4A). Matrigel was used to simulate the extracellular matrix, and the effect of CCAT1 on the invasion capacity of bladder cancer cell lines was analyzed by examining the number of cells that digested the matrix and passed through the polycarbonate membrane. Our study revealed that knock-down of CCAT1 weakened the invasion abilities of 5637 and T24 cells compared with the controls (P < 0.05, Figure-4B).

### Potential signaling pathways may be related to some miRNAs

**Figure 5:**
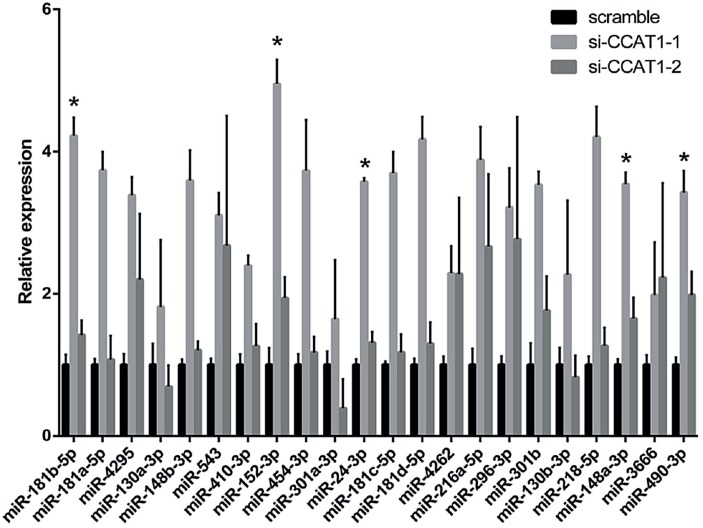
Differential expression levels of 22 miRNAs in T24 cells transfected with scramble, si-CCAT1-1 or si-CCAT1-2.

We further examined potential signaling pathways by predicting 22 miRNAs that were targets of CCAT1 using Starbase 2.0; the differential expression levels of the 22 miRNAs in T24 cells transfected with si-CCAT1-1, si-CCAT1-2 and scramble siRNA were detected by qRT-PCR. The results showed that miR-181b-5p, miR-152-3p, miR-24-3p, miR-148a-3p and miR-490-3p expression was significantly different in both the si-CCAT1-1 group and the si-CCAT1-2 group compared to the scramble siRNA control group (Figure-5).

## DISCUSSION

An estimated 430.000 new cases of bladder cancer occurred in 2012, making bladder cancer the ninth most common cancer worldwide (19). In recent decades, we have not been very successful at reducing the prevalence of bladder cancer for many reasons, such as smoking (20). Although we have observed a global reduction in bladder cancer mortality in recent years, a more positive trend is impossible without more effective treatment modalities or new treatments. However, the molecular mechanisms underlying the tumorigenesis and progression of bladder cancer are yet to be elucidated.

lncRNAs represent a novel class of noncoding RNAs that are longer than 200 nucleotides and do not have protein-coding potential. Recently, lncRNAs were found to be dysregulated and involved in various cancer biological processes, such as proliferation, apoptosis, mobility, and invasion (21, 22). However, the role and precise molecular mechanism of lncRNAs in cancer development and progression still remain elusive (11). In previous studies, CCAT1 was found to be involved in the development and progression of a variety of cancer types (11, 12, 23), but the functional effect of CCAT1 on the development of bladder cancer has not been reported.

In our study, we demonstrated that CCAT1 was aberrantly increased in human bladder cancer tissues relative to adjacent nontumor tissues. In addition, the expression of CCAT1 in 5637 and T24 cell lines further confirmed this observation. Additionally, chi-square analysis was performed to demonstrate the relationship between relative CCAT1 expression and clinical characteristics. Due to the positive correlation between CCAT1 and clinical characteristics, we also performed a cell proliferation assay, an apoptosis assay, a wound healing assay and a cell invasion assay. The results suggested that the downregulation of CCAT1 suppressed cell proliferation, migration and invasion, which meant that CCAT1 can promote the progression of bladder cancer. These results suggest that CCAT1 expression may be a potential prognostic marker for patients with bladder cancer.

Emerging evidence has indicated that lncRNAs function as competing endogenous RNAs (ceRNAs) via sponging miRNAs (24). We performed a search for miRNAs that had complementary base pairing with the lncRNA CCAT1 and identified 22 miRNAs with tight pairing. The expression levels of most miRNA were increased in knock-down cells, concordant with a possible function as competing endogenous RNA. In the cell functional test, we got coincident results between si-CCAT1-1 group and si-CCAT1-2 group, so we paid attention to the consistently upregulated miRNAs at first. Among these miRNAs, miR-181b-5p, miR-152-3p, miR-24-3p, miR-148a-3p and miR-490-3p may be related to the function of CCAT1. Particularly, all of these miRNAs have been proven to be related to the development of tumor malignancies in previous studies (25–30). However, whether CCAT1 can sponge some of the se miRNAs requires elucidation in future studies.

Several limitations exist in our study. The results of qRT-PCR revealed that not all bladder tissues have upregulated CCAT1 expression; thus, our study of bladder cancer cell lines has uncertainty, even though we performed our experiments on two types of bladder cancer cell lines with relatively high CCAT1 levels. Furthermore, our study demonstrated that CCAT1 has an effect on both proliferation and migration, so differentiated proliferation may have affected the migration experiments.

## CONCLUSIONS

In summary, our findings suggested that CCAT1 was abnormally increased in patients with bladder cancer and that high levels of CCAT1 expression were positively related to aggressive tumor characteristics. The downregulation of CCAT1 expression inhibited bladder cancer cell proliferation, migration and invasion. In addition, miR-181b-5p, miR-152-3p, miR-24-3p, miR-148a-3p and miR-490-3p may be related miRNAs that mediate the aforementioned functions of CCAT1. Taken together, our findings demonstrated that CCAT1 may serve as a potential prognostic marker and therapeutic target in bladder cancer.
